# Mapping a comprehensive assessment tool to a holistic definition of health for person-centred care planning in home care: a modified eDelphi study

**DOI:** 10.1186/s12913-023-10203-5

**Published:** 2023-11-16

**Authors:** A. Fowokan, J.L. Giosa, M. Saari, P. Holyoke

**Affiliations:** 1 SE Research Centre, SE Health, 90 Allstate Parkway, Suite 800, Markham, ON L3R 6H3 Canada; 2 School of Public Health Sciences, University of Waterloo, 200 University Avenue West, Waterloo, ON N2L 3G1 Canada; 3 Lawrence S. Bloomberg Faculty of Nursing, University of Toronto, 155 College Street, Toronto, ON M5T 1P8 Canada

**Keywords:** Home care, Comprehensive assessment, eDelphi, Health definition, Holistic health, interRAI, Person-centred

## Abstract

**Background:**

Researchers in the Netherlands proposed the Pillars for Positive Health (PPH) as a broadly encompassing health definition to support more realistic and meaningful care planning for people living with chronic disease and other life-long health conditions. The PPH was subsequently converted to the My Positive Health (MPH) spider web visualization tool. This study sought to identify opportunities for more person-centred care planning at the point of care in home care, using the MPH tool as a framework to link comprehensive assessment and dialogue-based goal-setting.

**Methods:**

A modified eDelphi method was used to conduct domain mapping with a purposively sampled expert panel (n = 25). The panel consisted of researchers, health care providers, older adults and caregivers. A two-stage eDelphi process was conducted, with each stage consisting of three survey rounds. In the first stage, participants were asked to map 201 elements of the interRAI Home Care (interRAI HC) comprehensive assessment tool to the six MPH domains or “No pillar of best fit”. The second stage focused on identifying opportunities to adapt or expand comprehensive assessment as it relates to the MPH domains.

**Results:**

In Stage 1, 189 of 201 elements reached consensus in domain mapping. These included: 80 elements for Bodily Functions, 32 for Daily Functioning, 32 for Mental Wellbeing, 24 for Quality of Life, 10 for Participation, and 1 for Meaningfulness. Ten elements were identified to have no pillar of best fit. The 12 elements that did not reach consensus in Stage 1 formed the basis for Stage 2, where expert panel participants proposed four new assessment elements in Meaningfulness and Participation and 11 additional descriptors across the six MPH domains. Of these, two elements and nine of the 11 descriptors reached consensus.

**Conclusion:**

Findings show that elements of the interRAI HC are oriented toward the physical, functional, and mental health domains. Consequently, complementary assessment elements and/or tools may be needed to support comprehensive assessment of ‘Meaningfulness’ and ‘Participation’ in person-centred home and community care. Additional descriptors may also be needed to aid communication regarding the understanding and application of MPH domains.

**Supplementary Information:**

The online version contains supplementary material available at 10.1186/s12913-023-10203-5.

## Background

How we define health has significant ramifications for care delivery across different settings [1]. The World Health Organization’s (WHO) definition of health as a state of complete physical, and social wellbeing, and not merely the absence of disease [2], is one of the most commonly referenced definitions [3]. Promulgated at the founding of the WHO in 1948 [2], its decision to include mental and social dimensions as integral components to healthy functioning, thus broadening the lens through which health was viewed, was at the time considered pioneering [4]. At present, however, the definition’s focus on completeness appears incongruous with current epidemiological realities related to chronic disease [5, 6]. For individuals managing chronic health conditions, the WHO’s definition does not provide a person-centred goal on which their health needs could be assessed. For the purposes of this study, being person-centred is defined as an approach to health and healthcare that seeks to promote dignity and respect, information sharing, participation in decision making and collaboration with healthcare stakeholders in the development, implementation and evaluation of healthcare delivery [7]. For health systems to plan and deliver services tailored to the unique needs of individuals, a person- and goal-centred definition of health is needed [6].

Recognizing the limitations of the WHO’s health definition, a multidisciplinary group of international healthcare experts attending a two-day conference in the Netherlands explicated a new vision of health that acknowledges its dynamic nature and underscores the individual’s ability to adapt to changing health states [8]. Health was subsequently conceptualized as “the ability to adapt and to self-manage” at the two-day conference [4]. This definition was later operationalized into six measurable dimensions – Bodily Functions (i.e., concepts relating to the physiological systems of the body e.g., physical functioning, pain, health symptoms etc.), Daily Functioning (i.e., concepts relating to an individual’s ability to perform basic and instrumental activities of daily living), Societal Participation (i.e., an individual’s involvement or connectedness to community or society), Quality of Life (i.e., concepts relating to an individual’s overall perception of and satisfaction with how things are in their life), Meaningfulness (i.e., concepts relating to an individual’s quest to find purpose or seek meaningful connections) and Mental Wellbeing (i.e., concepts relating to an individual’s mental and emotional wellbeing) – with 32 sub-dimensions [8]. This broad operational categorization was termed the Pillars for Positive Health (PPH) framework [8]. While the PPH framework is widely known and has been used in home and community care practice in the Netherlands, to the best of our knowledge, it has not been previously used in peer-reviewed home and community care research.

Home and community-based care provides an optimal backdrop for the implementation of a broader conceptualization of health. Persons receiving home care services have complex needs, often having multiple chronic medical conditions complicated by functional impairments and social frailty [9, 10]. Available evidence demonstrates that most older adults prefer to live, age, and receive care at home [11, 12], however a range of medical, functional and social factors can influence the need to transition to facility-based care [9, 13, 14]. Furthermore, goals of home-based care focus on promoting, maintaining, or restoring health while maximizing independence and minimizing the effects of disability and illness [15], thus necessitating a more comprehensive understanding of preferences, strengths and needs. The PPH framework, given its robustness, practicality, and ability to reflect current epidemiological realities, may be particularly well-suited to guiding the point-of-care assessment approach in home and community care.

Comprehensive assessment of client needs has been demonstrated to be an integral part of the care planning process in home care, as it supports the collation of health information which is used to develop individualized, person-centred plan aimed at improving overall health outcomes [16]. A recent scoping review identified that several comprehensive assessment tools have been developed to support holistic care planning in home care, including the interRAI HC [17]. However, findings from a recent survey showed that point-of care use of the interRAI HC may not align with intended use, including supporting care planning efforts within the home care sector in Ontario, Canada [18]. Reasons for this are not well understood, but it is plausible that the robustness of the interRAI HC from an assessment and documentation perspective, could make the process challenging for both the assessor and the client [18]. When utilized as designed as a tool to support care planning, client-level outcomes have been shown to improve [19].

An evidence-based approach to the successful integration of comprehensive assessment tools in the care planning process in home care is through the process of dialogue-based goal setting [20–22]. In a recent participatory research study on integrated care planning in home care, the PPH framework was recommended as a tool to guide the implementation of a person-centred, dialogue-based goal setting approach to care planning [23]. These findings guided leaders at a large Canadian home care organization to adopt the PPH framework as a definition of ‘life care’ – a person-centred principle of care that recognizes that the needs of people in community health care settings extend beyond physical and functional domains [23]. The PPH’s dimensions and sub-dimensions were subsequently converted to the My Positive Health (MPH) tool to support use in practice [24]. The MPH tool has the potential to support meaningful dialogue between point-of-care providers, older adults and caregivers and support the integration of life goals into the plan of care in community settings [23].

The overall goal of this study was to identify opportunities to facilitate more person-centred care planning at the point-of-care using the MPH tool as a framework to link comprehensive assessment and dialogue-based goal setting in home care. Specifically, the study aimed to: (1) map the elements of a standardized comprehensive assessment tool mandated for use in Ontario home care onto the six domains of the MPH tool, (2) identify opportunities to adapt or expand the MPH tool based on unmapped items from the comprehensive assessment, and (3) identify opportunities to adapt or expand comprehensive assessment to support life care, as defined by the MPH domains.

## Methods

### Study design

We used a modified eDelphi method for this study. The Delphi method was developed by the Rand Corporation and first described in a paper by Dalkey and Helmer (1963) [25]. The method adopts a systematic approach to consensus building by means of iteration with controlled feedback of the group’s opinion and the aggregation of responses to address differing viewpoints [26, 27]. The Delphi technique employs the use of quantitative and qualitative methods (mixed-methods design) to collect and analyze information from participants. Typically, a Delphi study is conducted through the following processes: (1) Identify an expert panel based on an established eligibility criteria [26, 28, 29]; (2) Develop a questionnaire containing elements that the expert panel are required to vote on or rate [27]; (3) Establish a consensus criteria by which level of agreement will be decided prior to commencement [30–32]; (4) Provide participants with anonymized summaries of the findings after each Delphi round, including participant’s own score or ranking, allowing participants to evaluate findings within the context of group responses [27, 29].

Two modifications to the traditional Delphi method were employed for this study. First, due to the geographical spread of the prospective expert panel as well as considerations for social distancing during the pandemic, the survey was conducted online using an eDelphi approach. Additionally, because the initial idea generation phase was replaced with pre-existing information (e.g., interRAI HC elements and MPH domains), this study was considered a modified eDelphi study. We conducted the eDelphi study over two stages. Stage 1 addressed objective 1, and Stage 2 addressed objectives 2 and 3.

### Delphi participant recruitment

Non-probability purposive sampling combined with snowball sampling [33] was adopted to recruit international experts who self-identified as fluent in English and familiar with the interRAI suite of assessment tools, comparable comprehensive assessment tools and/or the MPH framework. The process began by identifying names of individuals with the following perspectives: researcher, health and social care provider, older adults with interest or experience in home and community care and/ or caregivers. For those in the researcher, health, and social care provider groups, the study team first compiled a list of individuals within professional networks who met the recruitment criteria described above. These individuals were then sent a recruitment email with detailed information about the study from a member of the study team who had no prior working relationship with them. Those interested in participating in the expert panel were asked to reply to the recruitment email to communicate their interest in participation. For individuals in the client and caregiver groups, representatives of known older adult and caregiver support groups were contacted with detailed information about the study, asking interested parties to contact a member of the study team. Familiarity with the interRAI HC and/or the MPH tool was defined as firsthand experience with the interRAI HC assessment process in their capacity as clients or caregivers. Consent was obtained from participants electronically prior to the start of the first eDelphi survey. Participants who completed all stages of the eDelphi study were offered an optional $50 gift card as an honorarium.

### Stage 1

The first stage consisted of three survey rounds and sought to address the first study objective—to map the elements of a standardized comprehensive assessment tool mandated for use in Ontario home care (i.e., the interRAI HC assessment) onto the six domains of the MPH tool. All surveys were conducted online using Qualtrics software (2021; Provo, UT).

### InterRAI assessment

interRAI (www.interRAI.org) is a not-for-profit network of more than 135 researchers, clinicians, and policy experts from over 35 countries focused on the development and application of comprehensive assessment, screening, and care planning systems that identify and respond to the needs of vulnerable persons of all ages across the continuum of care. With over three decades of research experience resulting in over 1400 scientific publications, interRAI has developed assessment systems that have been adopted on a large-scale basis internationally across multiple settings including home and community care, long-term care and both community and in-patient mental health [34, 35]. Across Canada, interRAI tools have been mandated for use in home care and long-term care and, in the Province of Ontario, in in-patient mental health as well as community support services [36–38].

The interRAI HC assessment is part of the interRAI suite of assessment tools and is used to evaluate the care needs of home care clients [39]. The reliability and validity of the interRAI HC assessment tool has been established internationally [40–42]. The interRAI HC contains more than 300 elements used to assess the needs of home care clients across a range of domains to support care planning and monitoring efforts [39].

It is important to note that the interRAI HC manual recommends the adoption of a dialogue-based approach to the use of the interRAI HC as an assessment tool, thus aligning with the overarching study goals [43]. However, the outlined study goal seeks to provide a clearer, more accessible way to facilitate this, given known barriers to adoption cited previously. After detailed review of the interRAI HC, study investigators flagged 201 assessment items across 19 of 20 assessment categories that sought to directly assess an individual’s health status and excluded elements relating to identifying information, specific medication details or those deemed not directly relevant or tangential to the assessment of individual health status [43]. Assessment elements for expert panel consultation were then defined for ease of interpretation outside of the assessment context (see Table 1 for examples).

### Data collection

In the first survey, participants were presented with detailed information about the study, including study objectives, information about the interRAI HC and an image of the MPH tool that included descriptors for each of the six MPH domains (i.e., Bodily Functions, Daily Functioning, Societal Participation, Quality of Life, Meaningfulness, and Mental Wellbeing) [24]. Participants were then asked to match each of the interRAI HC assessment elements with one of the six MPH domains they deemed to be of best fit. A “No Pillar of Best Fit” option was also provided for assessment elements panel members determined to not fit into any of the six MPH domains. Consistent with best practices for Delphi studies, consensus was defined a priori and was set as 70% or greater agreement [31, 44, 45]. In each successive survey round, elements that reached consensus were documented and removed from the survey and participants were invited to respond to the survey again. To facilitate consensus-building, participants were provided with a summary file following each round that contained their individual responses and the summary of the overall expert panel responses from the preceding round (see supplemental file 1 for sample Stage 1 report). Each stage of the study was restricted to three rounds in line with Delphi best practices for consensus determination [46, 47].

### Stage 2

The second stage focused on identifying opportunities to adapt or expand the MPH tool based on unmapped items from the comprehensive assessment and identifying opportunities to adapt or expand comprehensive assessment to support life care, as defined by the MPH domains. This stage was also conducted over three survey rounds.

### Data collection

In round 1 of the second stage, MPH domains that were considered underrepresented by interRAI HC elements (defined a priori for this study as domains reflecting less than 1/12th of the total interRAI HC element list) and interRAI HC elements that did not reach consensus after stage 1 were considered. Participants were asked to propose suggestions for additional assessment elements and/or domain ideas. Panel responses on proposed new MPH domain ideas and assessment elements were then compiled and in survey round 2 participants were asked to rate their agreement with their inclusion using a 5-point Likert scale (i.e., 1- Strongly Disagree, 2- Disagree, 3- Neutral, 4, Agree, 5- Strongly Agree). As in Stage 1, consensus was defined as proposed MPH domain ideas or assessment elements rated 4 or greater by 70% or more of the Delphi participants. In round 3, after removing elements that reached consensus in round 2, participants were invited to respond to the survey again. To facilitate consensus-building, participants were provided a summary of findings from the previous rounds to inform their decision.

In both stages, the link to the survey was sent individually to each participant by email. Participants had three weeks to complete each survey. A reminder was provided to each participant who had not completed the survey after every seven days (i.e., days 7, 14 and 21). Individuals who did not complete the survey after the three-week period were considered to have opted out of the process and were not invited to participate in subsequent rounds.

### Data analysis

#### Analysis of Stage 1 surveys

Descriptive statistics (i.e., means and standard deviations for continuous variables and percentages and frequencies for categorical variables) were used to describe the demographic characteristics of eDelphi participants. To compare the demographic characteristics of eDelphi starters and completers, independent t-tests for continuous variables and chi-square analyses for categorical variables were conducted.

After each survey round in Stage 1, descriptive statistics (percentages) were used to summarize the distribution of responses for each assessment element matched to the MPH domains, and to identify elements that reached consensus after each survey round. Modes were used to identify the most frequently occurring response option for each interRAI HC assessment element.

#### Analysis of Stage 2 surveys

Qualitative data from survey 1 of Stage 2 regarding expert panel suggestions for additional assessment elements and additional MPH domain ideas were categorized thematically using Microsoft Excel for Microsoft 365 MSO version 2202. To enhance the reliability of this exercise, this process was conducted by AF and independently verified by all co-authors, first through asynchronous review, and then in a consensus-building meeting.

For surveys 2 and 3 of Stage 2, descriptive statistics (modes and percentages) were used to summarize panel members agreement ratings for additional assessment elements and MPH domain ideas and to identify suggestions that reached consensus after each round.

## Results

**Fig. 1:**
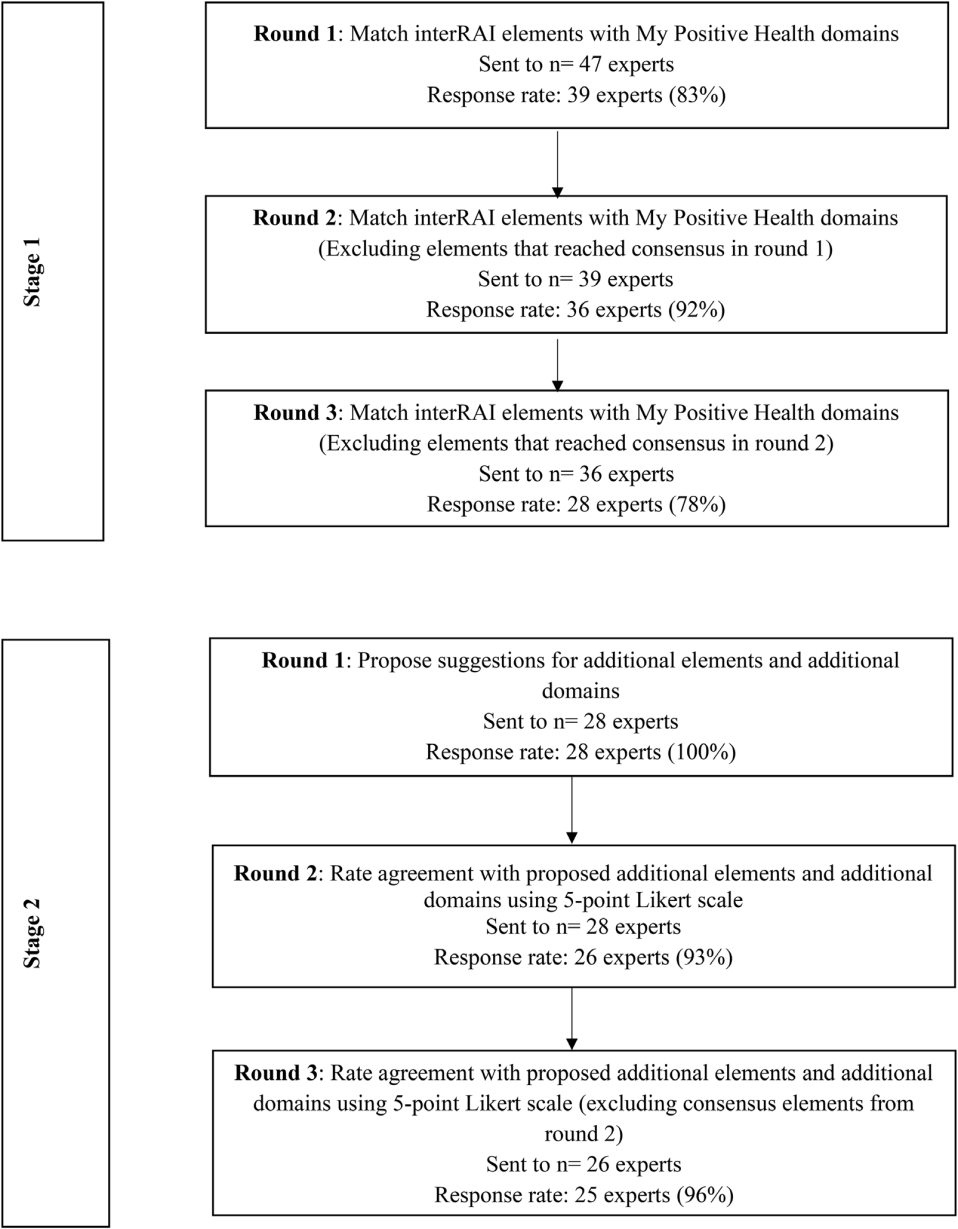
Flow Diagram Presenting the Steps in the Delphi Consensus Process

Forty-seven experts responded to the study recruitment email and were sent the link to survey 1 of the eDelphi study. Of those, 39 expert panel members completed the first survey and provided demographic information. In total, 25 expert panel members completed all six rounds (two stages) of the eDelphi study (Fig. 1). Demographic information describing characteristics of study starters and completers is provided in Table 1. No significant differences in demographic characteristics were observed between expert panel members who started and those who completed the full study (p > 0.05). Expert panel members included 33 females and 6 males representing a broad range of age groups: 31–40 (n = 9), 41–50 (n = 6), 51–60 (n = 6), 61–70 (n = 10), 71–80 (n = 7) and 81–90 (n = 1). A total of 22 experts identified with the researcher/health care provider perspective while 17 identified as caregivers and/or older adults. Geographic distribution of eDelphi participants are included in Table 1.

**Table 1:** Characteristics of Delphi Expert Panel

Participant Characteristics	Delphi Starters (n = 39)	Delphi Completers (n = 25)	P value
**Sex**			0.947
Female	33 (84.6%)	21 (84.0%)	
**Age Range**			0.946
31–40	9 (23.1%)	4 (16.0%)	
41–50	6 (15.4%)	4 (16.0%)	
51–60	6 (15.4%)	3 (12.0%)	
61–70	10 (25.6%)	9 (36.0%)	
71–80	7 (17.9%)	4 (16.0%)	
81–90	1 (2.6%)	1 (4.0%)	
**Perspective**			0.729
Researcher/Health Provider	22 (56.4%)	13 (52.0%)	
Caregiver/Older Adult	17 (43.6%)	12 (48.0%)	
**Location**			0.883
Ontario	33 (84.6%)	20 (80.0%)	
Other Canadian Province	5 (12.8%)	4 (16.0%)	
Outside Canada	1 (2.6%)	1 (4.0%)	

### Stage 1 results

#### Round 1 survey

Thirty-nine out of 47 (83% response rate) individuals who agreed to be part of the expert panel by email completed the first survey. In round 1 there was consensus on 84 (41.8%) of the 201 interRAI HC assessment elements among the expert panel members. Of these, 46 (54.8%) interRAI elements reached consensus in the Bodily Functions domain, 15 (17.9%) in Daily Functioning, 10 (11.9%) in Mental Wellbeing, 7 (8.3%) in Quality of Life, and 6 (7.1%) in Participation. No interRAI HC assessment elements reached consensus in the Meaningfulness domain or in No Pillar of Best Fit.

#### Round 2 survey

In round 2, expert panel members were presented with 117 non-consensus interRAI HC assessment elements from survey 1. Thirty-six out of 39 (92% response rate) expert panel members completed survey 2. Forty-nine (41.9%) of 117 assessment elements reached consensus across expert panel members. Of these, 18 (36.7%) elements reached consensus in the Bodily Functions domain, 13 (26.5%) in Mental Wellbeing, 6 (12.2%) in Quality of Life, 5 (10.2%) in Daily Functioning, and 2 (4.1%) in Participation. Five (10.2%) elements reached consensus in No Pillar of Best Fit. No assessment elements reached consensus in the Meaningfulness domain.

#### Round 3 survey

In round 3, the expert panel were presented with the remaining 68 interRAI HC assessment elements which had not yet reached consensus. Twenty-eight of thirty-six (78% response rate) expert panel members completed survey 3. Fifty-three (77.9%) of the 68 assessment elements were found to reach consensus. Of these, 16 (30.2%) reached consensus in Bodily Functions, 12 (22.6%) in Daily Functioning, 11 (20.8%) in Quality of Life, 9 (17.0%) in Mental Wellbeing, 2 (3.8%) in Participation and 1 (1.9%) in Meaningfulness. Five elements (7.4%) reached consensus in No Pillar of Best Fit.

**Fig. 2:**
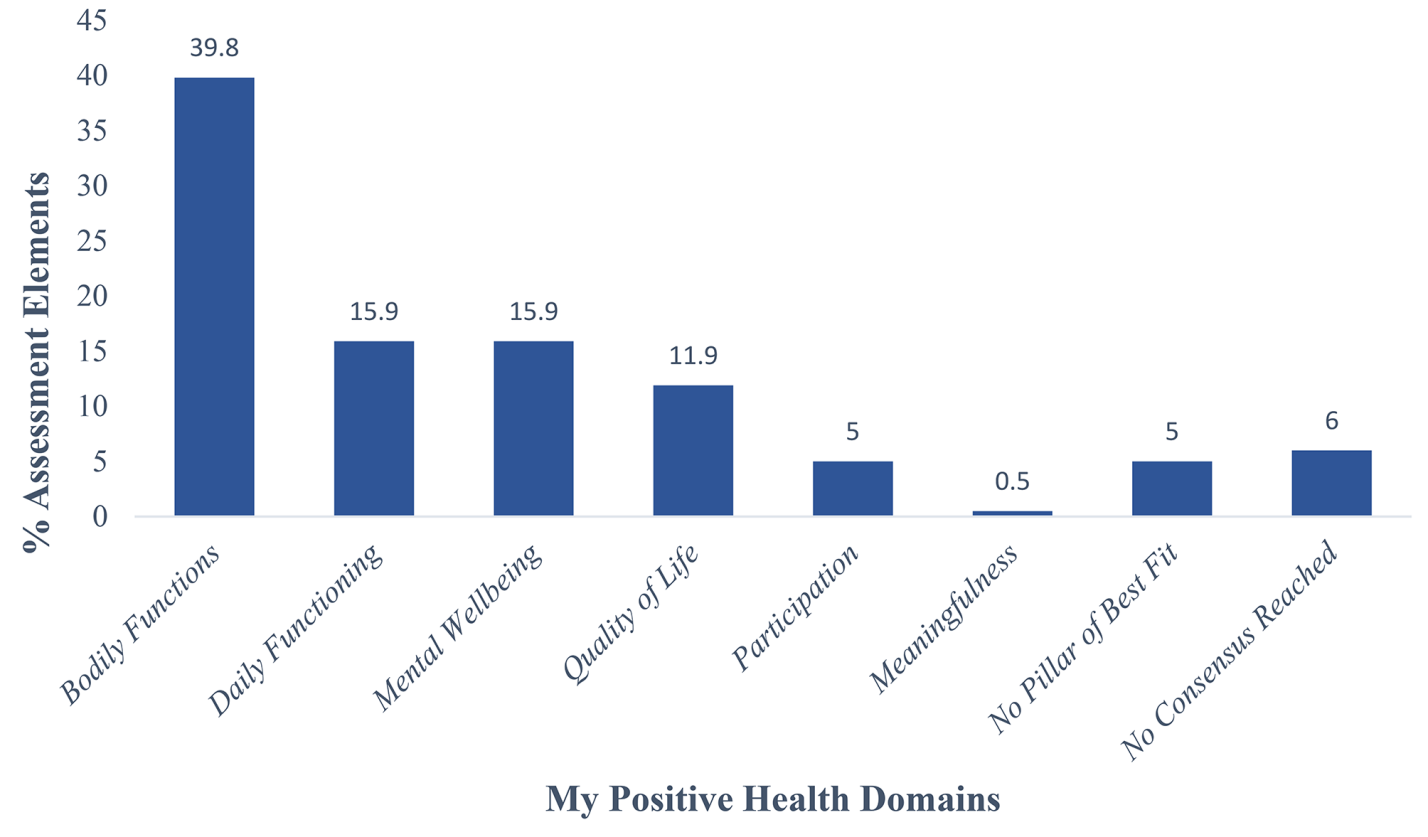
List of Assessment Elements that Reached Consensus by Specific My Positive Health Domain

Over the course of the three surveys conducted in Stage 1 of the eDelphi, 189 of 201 assessment elements reached consensus. Of these, 80 (39.8%) reached consensus in the Bodily Functions domain, 32 (15.9%) in Daily Functioning, 32 (15.9%) in Mental Wellbeing, 24 (11.9%) in Quality of Life, 10 (5.0%) in Participation, and 1 in Meaningfulness (Fig. 2; a specific list of assessment elements that reached consensus is provided in Table 2). Ten (5.0%) assessment elements reached consensus in No Pillar of Best Fit, while 12 (6.0%) assessment elements did not reach consensus in Stage 1 and formed the basis for Stage 2 of the eDelphi study (Fig. 2).

**Table 2:** List of interRAI HC Assessment Elements that Reached Consensus by Specific Pillar

#	Bodily Functions (total consensus = 80)	Daily Functioning (total consensus = 32)	Participation (total consensus = 10)	Quality of Life (total consensus = 24)	Meaningfulness (total consensus = 1)	Mental Wellbeing (total consensus = 32)	No Pillar of Best Fit (total consensus = 10)
1	Person’s hearing ability	Person’s ability to prepare their own meals (e.g., planning meals, assembling ingredients)	Person demonstrated reduced social interactions	Person’s home is in a state of disrepair (e.g., hazardous clutter)	Person reports little interest or pleasure in activities normally enjoyed	Person’s short-term memory is OK (e.g., person appears to be able to recall after 5 min)	Person’s gender
2	Person’s vision (i.e., person’s ability to see close objects in adequate light using customary visual appliances for close vision)	Person’s ability to take part in housework (e.g., doing dishes, dusting)	Person’s participation in social activities of long-standing interest	Person lives in squalid conditions (e.g., infestation)		Person sometimes can’t “think straight” or sometimes struggles to concentrate	Where the assessment is being done (e.g., private home, assisted living setting, hospital, care home)
3	Time taken for person to do a 4-metre walk	Person’s ability to independently manage their finances (e.g., bills are paid, cheque book is balanced)	Person paid a visit to, or was visited by long-standing social relation or family member	Person’s home has inadequate heating/ cooling		Person is easily distracted (e.g., has episodes of difficulty paying attention, gets sidetracked)	Whether the person identifies as Indigenous (e.g., First Nations, Métis, Inuit)
4	Distance walked by person (with necessary support equipment)	Person’s ability to manage medication use (e.g., remembering to take medication, opening bottles, taking correct dosage)	Person interacted with long-standing social relation or family member (e.g., telephone or email)	Person feels unsafe and might be at risk of violence within or outside their home		Person’s mental function varies over the course of the day	Person’s primary language (e.g., English, French)
5	Person’s pattern of bladder continence	Person’s ability to use the phone (e.g., make and receive calls)	Amount of time person spends alone during the day (i.e., morning and afternoon)	Person lives in apartment or house that has been made accessible for persons with disabilities		Person has significant changes in usual level of mental functioning (e.g., restlessness, lethargy, altered perceptions of their environment)	Time since event or problem that led to person’s deterioration
6	Person’s pattern of bowel continence	Person’s ability to shop for household elements (e.g., selecting elements and paying for food and household elements)	Person has strong and supportive relationship with family	Person can go grocery shopping without assistance		Person was diagnosed with anxiety	Person’s marital status
7	Person was diagnosed with hip fracture	Person’s ability to transport themselves (e.g., can travel by public transportation or get in and out of house and vehicles)	Person stopped participating in activities of interest	Person is experiencing financial constraints which may impact their health or safety (e.g., made trade-offs among purchasing adequate food, shelter, clothing, prescribed medications)		Person was diagnosed with depression	Person’s family or close friends report feeling overwhelmed by person’s illness
8	Person was diagnosed with other fracture (i.e., any other fracture other than a hip bone fracture)	Person’s ability to bathe independently (e.g., person was able to transfer in and out of tub/ shower, bathe all parts of the body including: arms, upper and lower legs, chest and abdomen)	Number of days person went out of the house	Person’s living situation (e.g., independent/ semi-independent living, mental health residence, group home, hospital/ unit, homeless, correctional facility)		Person shows signs of abnormal thought processes (e.g., loosening of associations, thought blocking, flight of ideas)	Time since person’s last hospital stay
9	Person was diagnosed with hemiplegia	Person’s personal hygiene (e.g., combing hair, brushing teeth)	Person’s interest in social, religious, occupational or other preferred activities changed in the last 90 days	Person’s living situation over the last 5 years (e.g., prior stay in a residential care facility, board & care home, hospital/ unit)		Person shows signs of delusions (e.g., fixed false beliefs)	Person’s primary informal helper expresses distress, anger, depression
10	Person was diagnosed with paraplegia	Person’s ability to dress the upper body	Person has an informal helper (e.g., informal caregiver)	Person made nonverbal expressions demonstrating a lack of pleasure in life (e.g., anhedonia)		Person shows signs of hallucinations (e.g., false sensory perceptions)	Number of hours of informal care and active monitoring received by person in the last 3 days
11	Person was diagnosed with coronary heart disease	Person’s ability to dress the lower body		Person experienced a major stressful event in the last 90 days (e.g., episode of severe personal illness, death or illness of close member)		Person’s procedural memory is OK (e.g., person can do several tasks in a row without prompts)	
12	Person was diagnosed with chronic obstructive pulmonary disease	Person’s ability to cleanse self after toilet use or incontinence		Person has had one or more care goals met in the last 90 days		Person’s situational memory is OK (e.g., they can recognize caregiver’s names, faces of frequently encountered people, location of regularly visited places)	
13	Person was diagnosed with congestive heart failure	Change in person’s activities of daily living (e.g., bathing, showering, personal hygiene etc.) in the last 90 days		Person’s residential/ living status after discharge (e.g., private home, assisted living, hospital/ unit, care facility)		Person has episodes of disorganized speech	
14	Person was diagnosed with pneumonia	Person takes medications as prescribed by physician		Person made repetitive non-health-related concerns (e.g., seeking attention/ reassurance regarding schedules, meals, laundry and relationships)		Person’s decision making abilities are different compared to 90 days ago (e.g., abilities improved or declined)	
15	Person was diagnosed with urinary tract infection in last 30 days	Person demonstrates unusually poor hygiene		Person demonstrated sad, pained, or worried facial expressions (e.g., furrowed brows, constant frowning)		Person demonstrated persistent anger with self or others (e.g., easily annoyed, anger at care received)	
16	Person was diagnosed with cancer	Person’s skills for daily decision making (e.g., person is independent in decision making or is impaired in decision making)		Person was fearful of family member or close acquaintance		Person demonstrated unrealistic fears (e.g., fear of being abandoned, being left alone or being with others)	
17	Person was diagnosed with diabetes mellitus	Person resisted care (e.g., taking medications/ injections, activities of daily living assistance)		Person experienced neglect, abuse, or mistreatment		Person made recurrent statements that something terrible is about to happen	
18	Person has a recent history (e.g., last 30 days) of falling	Person drove car (vehicle) in last 90 days		Person expresses feelings of loneliness		Person reports feeling anxious, restless or uneasy	
19	Person experiences dizziness	Person is able to have grocery delivered to their home		Person’s self-reported health		Person reports feeling sad, depressed, or hopeless	
20	Person demonstrates an unsteady gait	Person’s overall self-sufficiency has changed significantly in the last 90 days		Person was physically restrained (e.g., limbs restrained to chair)		Person was diagnosed with Alzheimer’s disease	
22	Person experiences chest pain	Person’s stated goals of care		Informal caregiver lives with person		Person was diagnosed with dementia other than Alzheimer’s	
22	Person has difficulty clearing airway secretions	Person’s ability to move on and off toilet or commode		Person’s informal helper(s) is unable to continue caring		Person was diagnosed with bipolar disorder	
23	Person experiences symptoms of acid reflux (i.e., regurgitation of acid from stomach to throat)	Person’s ability to eat and drink (including other means of intake of nourishment by other means such as tube feeding)		Person has limited access to home/ rooms (e.g., unable to climb stairs)		Person was diagnosed with schizophrenia	
24	Person experiences constipation (i.e., no bowel movement in 3 days or difficult passage of hard stool)	Person’s primary means of movement indoors		Person has access to emergency assistance		Person’s ability to be understood (e.g., ability to express or communicate requests, needs, opinion and urgent problems and to engage in social conversation)	
25	Person experiences diarrhea	Person has been told to limit or stop driving				Person’s ability to understand others (i.e., ability to understand verbal information)	
26	Person experiences vomiting	Person’s conditions/ diseases make cognitive issues, activities of daily living, mood, or behaviour patterns unstable				Person cried or experienced tearfulness	
27	Person experiences symptoms of aspiration (i.e., coughing or wheezing after eating)	Person’s level of tobacco use (e.g., smokes tobacco daily)				Person wanders around frequently (e.g., moved with no rationale)	
28	Person has symptoms of fever	Person’s level of alcohol intake (i.e., number of drinks in any ‘single sitting’ in last 14 days)				Person demonstrated verbally abusive behaviour (e.g., threatened others, screamed at others etc.)	
29	Person has gastrointestinal or genitourinary bleeding	Decrease in amount of food/ fluid usually consumed by person				Person demonstrated physically abusive behaviour (e.g., others were hit, shoved)	
30	Person has symptoms of peripheral edema	Person ate one or fewer meals (i.e., or at least 2 over the last 3 days)				Person demonstrated disruptive or socially inappropriate behaviour (e.g., made disruptive sounds or noises)	
31	Person has symptoms of dyspnea (i.e., shortness of breath)	List of all medications taken by person				Person demonstrated sexually inappropriate behaviour or disrobed in public	
32	Intensity of pain experienced by person (e.g., mild, moderate, severe)	Person received care from a care professional in the last 7 days (e.g., home health aide, home nurse, physical/ occupational therapy, speech-language pathology)				Person reports feelings of conflict or anger with family/ friends	
33	Consistency of pain experienced by person (e.g., single episode, intermittent, constant)						
34	Person experienced breakthrough pain (e.g., sudden, acute flare-ups)						
35	Person’s height and weight						
36	Person’s degree of weight loss (i.e., 5% or more in last 30 days, or 10% or more in last 180 days)						
37	Person’s fluid output exceeds input						
38	Person has broken, fragmented, loose, non-intact natural teeth						
39	Person reports having dry mouth						
40	Grade (description) of person’s most severe pressure ulcer						
41	Person has a skin ulcer other than pressure ulcer						
42	Person has major skin problems (e.g., lesions, 2nd or 3rd degree burns)						
43	Person has skin tears or cuts						
44	Person has other skin conditions or changes in skin condition (e.g., bruise, rash, eczema)						
45	Person has foot problems (e.g., bunions, hammertoes, overlapping toes, structural problems, infections, ulcers)						
46	Person is allergic to any drug						
47	Person’s ability to walk up a full flight of stairs (e.g., manage 12–14 stairs)						
48	Person’s ability to walk around indoors (i.e., how person walks between locations on same floor)						
49	Person’s ability to move around (walking or wheeling) indoors						
50	Person’s ability to move around in bed (i.e., how person moves to/ from lying position, turns from side to side)						
51	Person uses urinary collection device (i.e., excluding pads/ briefs)						
52	Person uses pads or briefs for incontinence						
53	Person was diagnosed with Parkinson’s disease						
54	Person was diagnosed with quadriplegia						
55	Person was diagnosed with stroke/ cerebrovascular accident						
56	Person has difficulty moving self to standing position unassisted						
57	Person has difficulty turning around when standing						
58	Person has difficulty falling/ staying asleep						
59	Person is experiencing acute episode or flare-up of a recurrent or chronic problem						
60	Person shows signs of dehydration						
61	Person’s fluid intake is less than 1,000ml/ day						
62	Person wears denture(s)						
63	Person reports experiencing difficulty chewing						
64	Person has a history of prior pressure ulcer						
65	Person made repetitive health complaints (e.g., persistently seeking medical attention, incessant concern with body functions)						
66	Total hours of physical activity person participated in						
67	Person’s health care provider believes person is able to improve levels of physical function						
68	Person was diagnosed with multiple sclerosis						
69	Number of falls experienced by the person in the last 90 days						
70	Person experiences excessive sleep that interferes with normal functioning						
71	Person experiences fatigue (e.g., inability to complete normal daily activities)						
72	Frequency with which person complains or shows evidence of pain						
73	Person reports that pain control is adequate to the level of pain experienced						
74	How person consumes food (e.g., normal, only pureed solids, use of abdominal feeding tube)						
75	Person has had blood pressure measured in last year						
76	Person has had colonoscopy test in last 5 years						
77	Treatments received or scheduled by person (e.g., chemotherapy, intravenous medication, wound care)						
78	Person visited hospital, emergency room, or physician visit in the last 90 days						
79	Person had an inpatient acute hospital visit with overnight stay in the last 90 days						
80	Person had a physician or authorized assistant/ practitioner visit in the last 90 days						

### Stage 2

#### Round 1 survey

The expert panel members provided a range of suggestions for potential assessment elements for the two MPH domains (i.e., Meaningfulness and Participation) considered underrepresented by interRAI HC elements. After coding, four main themes for each domain were identified from the suggestions provided by the expert panel. For Meaningfulness, the following additional areas for assessment were identified: “person has thriving social support networks or feels included as part of a community”, “person’s ability to volunteer, make positive impact in their communities, or feels part of something bigger”, “person practices mindfulness, is self-aware, or has a spiritual life”, and “person has goals and purpose (i.e., work, career, vocations, occupations)”. For Participation, the following additional areas for assessment were identified: “person’s social and demographic information”, “person has thriving social support networks or feels included as part of a community”, “person’s ability to engage in activities of interest”, and “person’s emotional health and wellbeing”. Notably, one theme, “person has thriving social support networks or feels included as part of a community” was identified in both underrepresented MPH domains. Upon deliberation, the study team agreed to let the expert panel decide through the process of consensus in subsequent surveys, as to which MPH domain the duplicated theme best fit into. Detailed information on suggestions provided by expert panel in each theme is included in Supplemental File 2.

**Fig. 3:**
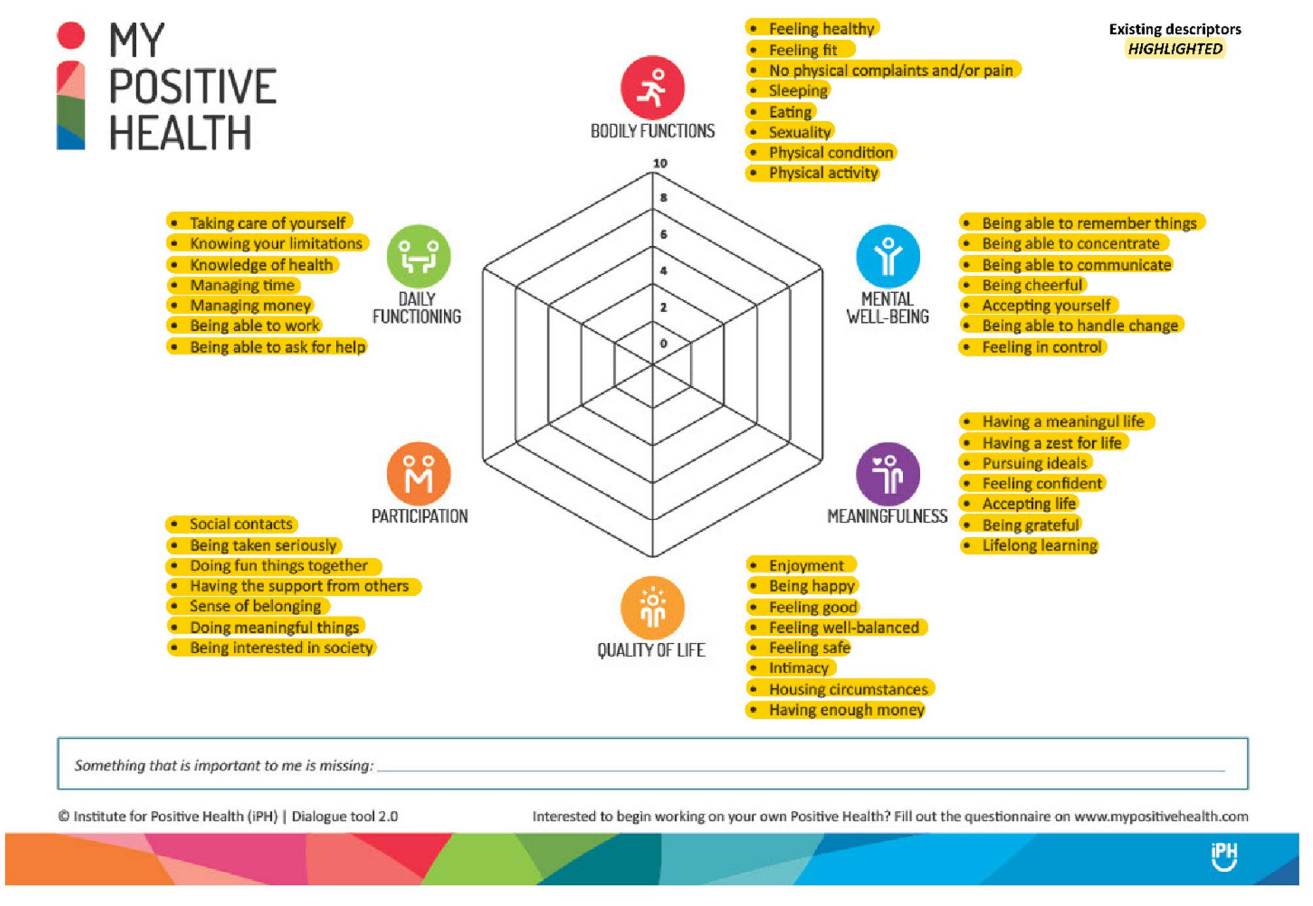
The My Positive Health Spider Web Visualization Tool with Descriptors

The expert panel also provided a range of suggestions about potential new MPH domain ideas for the interRAI HC elements that did not reach consensus in Stage 1 of the eDelphi study. Upon detailed review and categorization by the study team, the suggestions provided appeared to be additional descriptors (i.e., the highlighted bullet points for each pillar in Fig. 3) for the existing six MPH domains as opposed to entirely new MPH domains. A total of 11 additional descriptors across the six MPH domains were identified: 5 additional descriptors for Bodily Functions, 2 for Daily Functioning, and 1 each for Participation, Quality of Life, Meaningfulness and Mental Wellbeing.

#### Round 2 survey

In round 2 of the second stage of the eDelphi study, a total of three suggested assessment elements reached consensus. The expert panel agreed that “person has goals and purpose (i.e., work, career, vocations, occupations)” fit in Meaningfulness. Additionally, the panel agreed that “person has thriving social support networks or feels included as part of a community” and “person’s ability to engage in activities of interest” fit in Participation.

For the proposed additional descriptors, a total of 9 out of the 11 reached consensus for describing at least one of interRAI HC elements that were the subject of Stage 2.

#### Round 3 survey

At the end of survey 3, one additional proposed assessment element reached consensus in Meaningfulness, while no assessment elements reached consensus in Participation. The duplicated assessment item, which previously reached consensus in Participation in round 2 (i.e., person has thriving social support networks or feels included as part of a community) also reached consensus in Meaningfulness in round 3.

**Fig. 4:**
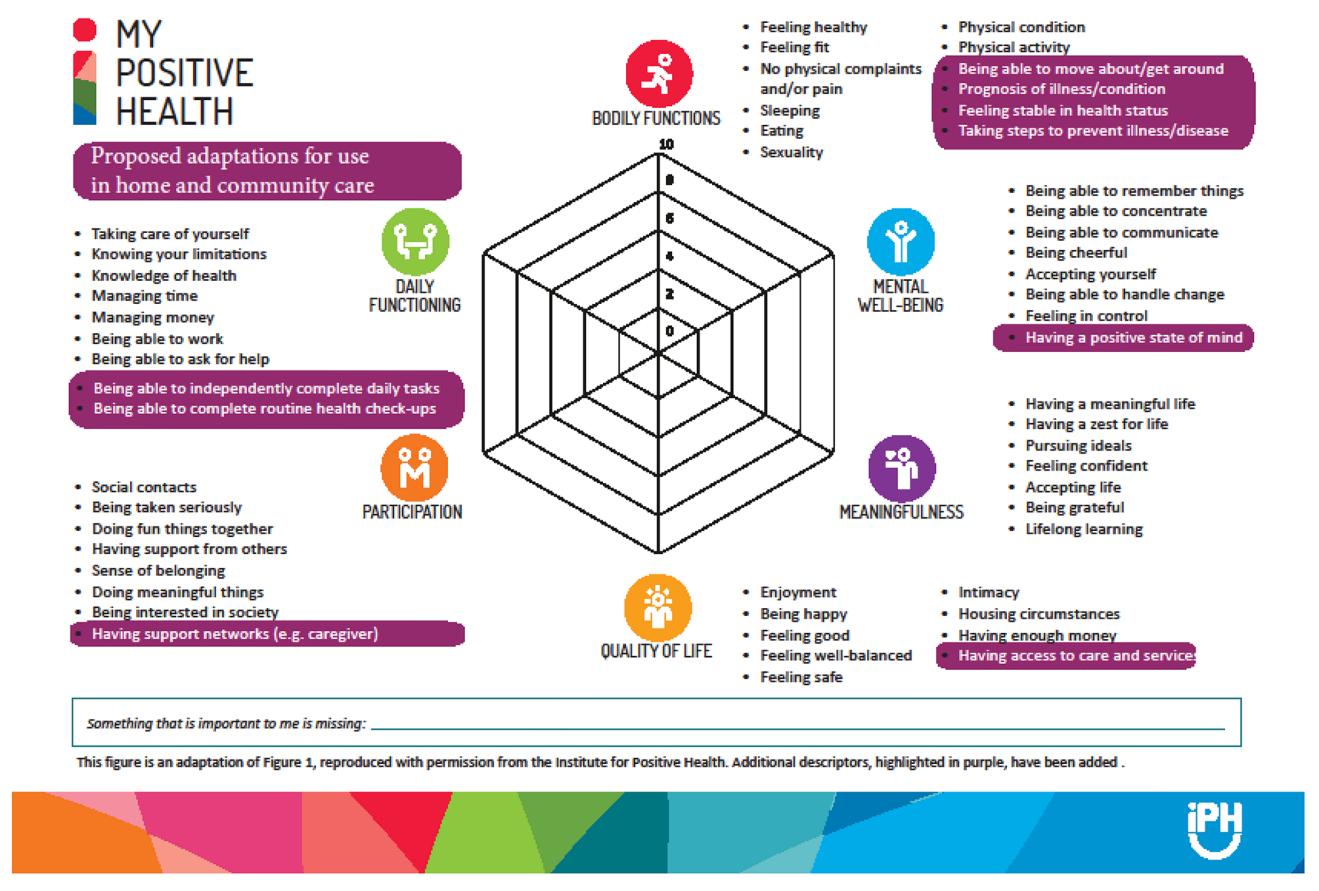
Expanded My Positive Health Image to include Phase 2 Findings

At the end of round 3, 9 of the 11 proposed additional descriptors in Stage 2 reached consensus. This information was subsequently used to recreate the MPH figure to incorporate the consensus additional descriptors (Fig. 4). A detailed summary of the list of proposed elements and additional descriptors for underrepresented MPH domains and non-consensus assessment elements are included in Supplemental File 3.

## Discussion

This study explored opportunities to improve person-centred goal setting at the point-of-care in home care, using the MPH as a framework to link comprehensive assessment and dialogue-based goal setting. Through a modified eDelphi process, investigators mapped the items from a comprehensive assessment tool mandated for use in Ontario home care to the six domains of the MPH and explored opportunities to adapt or expand comprehensive assessment elements and/or MPH domains. Findings from this study showed that 189 of the 201 interRAI HC elements successfully mapped to the six MPH domains, with Bodily Functions, Daily Functioning and Mental Wellbeing being the most represented of all six domains. A total of 10 elements reached consensus in No Pillar of Best Fit, while the remaining 12 elements did not reach consensus. Similarly, additional assessment elements and MPH descriptors, considered important to health assessment and useful in clarifying the underlying aspect of each MPH domain, were proposed. Taken together, these findings provide valuable insight into the potential for integration of the MPH framework with the interRAI HC comprehensive assessment tool to support more individualized and holistic care planning at the point-of-care in home care.

The expert panel reached agreement on mapping 94% of the assessment elements drawn from the interRAI HC to the six domains of the MPH. Furthermore, the panel did not identify any additional domains which should be added to the framework. This level of agreement highlights the potential use of this framework to help structure comprehensive and person-centred assessment conversations around a broader definition of health, while building on existing international best practices in standardized assessment of home care client needs. Taking advantage of the MPH or comparable frameworks to provide a clear structure to guide a conversational assessment, care planning and goal setting approach, may facilitate the delivery of more individualized care which addresses the range of care needs across population groups. An approach like this is aligned with the intended assessment approach outlined by interRAI in their assessment manuals but is not always seen in practice [18, 43].

There was a disproportionate mapping of assessment elements to the Bodily Functions, Daily Functioning and Mental Wellbeing domains, leaving MPH domains of Meaningfulness and Participation underrepresented. Participants were asked to suggest supplementary assessment elements to address potential gaps in the Meaningfulness and Participation domains. While the expert panel members proposed a range of suggestions, only two additional elements achieved consensus in both these domains. This may suggest that expert panel members found it challenging to propose assessment elements in these domains or that members did not have a unified understanding or definition of Meaningfulness and Participation, making it difficult to come to consensus. This is particularly evident given the duplicative assessment item which was proposed and reached consensus in both domains. These findings may also be reflective of the entrenched nature of the conventional bio-medical model of health across health and social care settings, thus placing limits on one’s ability to visualize health outside of known medically focused health domains [48]. The resulting outcome of interRAI HC assessment elements mapping most heavily to physical and mental health domains may also reflect a bias in participants towards assessment of health needs in areas where health and social care services may be available to address issues identified; however, future research is needed to confirm this.

Our findings are consistent with other comparable studies conducted in the older adult population. In a systematic review that sought to map the domains of 67 commonly used assessment tools to evaluate frailty in older adults, it was observed that while all the tools included assessment elements in the biological domains, only nine tools included elements assessing social connections and social support, despite known links on the importance of these factors towards the conceptualizations of frailty [49]. In another systematic review that used gap map methods to appraise the literature regarding assessment of health and social support services to support functional ability in older adults, it was found that of 528 studies reviewed, only a few studies assessed domains such as social participation, financial security, ability to maintain relationships and communication [50]. However, most of the studies included assessed functional ability within domains related to physical and mental capacity such as mobility, mental function and neuromusculoskeletal function [50]. Consequently, there is a need for the development of new assessment tools, or the augmenting of existing assessment tools to ensure the propagation of a holistic, person-centred model of care planning, particularly for older adults whose health needs tend to be complex and multifaceted.

### Clinical implications

This study has implications for comprehensive care planning in home and community care. As demand rises for models of care that support more integrated health and social care service delivery [12], comprehensive and person-centred care practices are necessary to maximize needs-based care opportunities. Findings from this study indicate that the interRAI HC tool supports a full investigation of physical and mental health, but complementary assessment elements and/or tools may be required to support assessment of ‘life care’ according to the MPH; namely Participation and Meaningfulness domains. It is important to note that the MPH was not designed through a clinical assessment lens, which often prioritizes definitive inclusion criteria for each domain that is to be assessed. Results suggest that to support integration of an expanded definition of health into existing care planning practices, additional descriptors for each MPH domain may be needed to enhance understanding, interpretation of and differentiation between the MPH for providers, older adults, caregivers and researchers. For example, one of the suggested assessment elements in Stage 2 of the eDelphi method reached consensus in two MPH domains. Additional research is needed to explore further delineation opportunities among the domains.

Next steps for this research will involve co-designing tools and resources to support dialogue-based comprehensive assessment and goal setting around ‘life care’ needs. The co-design work will be completed in partnership with home care clinicians, leveraging study findings to integrate the interRAI HC, MPH domains, additional descriptors and complementary assessment tools into a person-centred care planning process at the point-of-care in home care.

### Strengths and limitations

While the MPH allowed us to categorize the interRAI assessment elements within holistic health domains, it should be noted that the MPH was developed in the Netherlands using the Dutch language and later translated to English. It is possible that aspects of the Dutch language could contain words that cannot be adequately translated into the English language leading to potential biases. While we sought to recruit participants with firsthand experience of the interRAI HC and/or MPH framework, there were no formal inclusion criteria to ensure all participants were starting with the same knowledge base, which may have influenced the study findings. As the majority of our expert panel members were from a single Canadian province, the generalizability of study findings may be limited, given differences in how care is delivered across provinces and across countries. However, as the interRAI HC is used across Canada and in several countries, there is potential to validate this mapping exercise in other jurisdictions using similar methods. The strengths of this study include use of the Delphi method, which allowed for the engagement of a diverse group of experts across a range of perspectives critical to person-centred care planning in home care – older adults and caregiver, health and social care providers, and researchers. Another strength of this study is that the findings can be adopted to potentially expand the clinical utility of the interRAI HC at the point-of-care, building on existing and mandated standardized comprehensive assessment practices in Ontario and elsewhere.

## Conclusion

Using an eDelphi approach which included experts with a range of perspectives, we found that most interRAI HC assessment elements mapped onto MPH domains representing physical and mental health, with domains representing social needs like participation and meaningfulness agreed on to be underrepresented by assessment elements. This led to the proposal of new assessment elements and domain descriptors to expand the scope of comprehensive assessment and provide greater clarity between the domains representing ‘life care’ needs through an expanded definition of health. These findings will be used to co-design a dialogue-based approach to comprehensive assessment and goal setting around ‘life care’ needs that has the potential to optimize the care planning process for older adult clients, caregivers and home care providers through a more person-centred lens.

### Electronic supplementary material

Below is the link to the electronic supplementary material.

Supplemental File 1: Sample Stage 1 Report

Supplemental File 2: Summary of Proposed Assessment Elements for Underrepresented My (i.e., Meaningfulness and Participation) Positive Health Pillars *note top bolded row is summary statement of all similar grouped suggestions below

Supplemental File 3: List of Proposed Assessment Elements and Proposed Additional Descriptors for Underrepresented My Positive Health domains and non-consensus assessment elements, respectively

## Data Availability

Data used in this study can be made available upon reasonable request from the corresponding author.
