# Supplementary material for: Mapping a comprehensive assessment tool to a holistic definition of health for person-centred care planning in home care: a modified eDelphi study
Source: BMC Health Serv Res. 2023 Nov 16;23:1268. doi: 10.1186/s12913-023-10203-5 (PMC10655331; doi:10.1186/s12913-023-10203-5)
Supplement: Supplementary file 1 — Supplemental File 1: Sample Stage 1 Report [file 12913_2023_10203_MOESM1_ESM.docx]

**Supplemental File 1: Sample Stage 1 Report**

Dear xxxx,

Thank you for participating in the Stage [1], Survey [1] of the eDelphi study.

84 of the 201 assessment elements reached consensus and are detailed in a separate report.

117 assessment elements did NOT reach consensus in survey 1 and require further consideration by members of the expert panel in survey 2.

Below you will find a summary of survey 1 results for these 117 assessment elements. You will note that for each assessment element, we have listed your individual response, as well as the overall distribution of expert panel responses.

Please have this report open on a second screen or printed for reference as you complete survey 2.

| **Item** | **Your Response** | **Distribution of expert panel responses (most frequent bold)** |
| --- | --- | --- |
| Person's gender | Mental wellbeing | Bodily functions= 13%  Participation = 13%  Quality of Life = 3%  Meaningfulness= 15%  Mental wellbeing= 5%  **No Pillar of Best Fit= 51%** |
| Person's marital status | Quality of life | Participation = 26%  Quality of Life = 21%  Meaningfulness= 8%  **No Pillar of Best Fit= 46%** |
| Where the assessment is being done (e.g., private home, assisted living setting, hospital, care home) | No Pillar of Best Fit | Daily functioning = 13%  Participation = 8%  Quality of Life = 23%  Mental wellbeing= 3%  **No Pillar of Best Fit= 54%** |
| Person's stated goals of care | Daily functioning | **Daily functioning = 33%**  Participation = 8%  Quality of Life = 28%  Meaningfulness= 18%  Mental wellbeing= 3%  No Pillar of Best Fit= 10% |
| Person's living situation (e.g., independent/ semi-independent living, mental health residence, group home, hospital/ unit, homeless, correctional facility) | Quality of life | Daily functioning = 15%  Participation = 5%  **Quality of Life = 67%**  Meaningfulness= 3%  No Pillar of Best Fit= 10% |
| Time since person's last hospital stay | No Pillar of Best Fit | Bodily functions= 31%  Daily functioning = 13%  Participation = 3%  Quality of Life = 13%  Mental wellbeing= 3%  **No Pillar of Best Fit= 39%** |
| Whether the person identifies as Indigenous (e.g., First Nations, Métis, Inuit) | No Pillar of Best Fit | Bodily functions= 3%  Participation = 28%  Quality of Life = 5%  Meaningfulness= 5%  Mental wellbeing= 5%  **No Pillar of Best Fit= 54%** |
| Person's primary language (e.g., English, French) | Daily functioning | Daily functioning = 13%  Participation = 21%  Quality of Life = 3%  Mental wellbeing= 8%  **No Pillar of Best Fit= 56%** |
| Person's living situation over the last 5 years (e.g., prior stay in a residential care facility, board & care home, hospital/ unit) | Participation | Daily functioning = 8%  Participation = 13%  **Quality of Life = 67%**  Meaningfulness= 3%  No Pillar of Best Fit= 10% |
| Person's skills for daily decision making (e.g., person is independent in decision making or is impaired in decision making) | Mental wellbeing | Bodily functions= 5%  **Daily functioning = 67%**  Participation = 3%  Quality of Life = 8%  Mental wellbeing= 18% |
| Person's procedural memory is OK (e.g., person can do several tasks in a row without prompts) | Mental wellbeing | Bodily functions= 5%  Daily functioning = 36%  Participation = 3%  Meaningfulness= 3%  **Mental wellbeing= 54%** |
| Person's situational memory is OK (e.g., they can recognize caregiver's names, faces of frequently encountered people, location of regularly visited places) | Mental wellbeing | Bodily functions= 3%  Daily functioning = 23%  Participation = 5%  Quality of Life = 5%  Meaningfulness= 3%  **Mental wellbeing= 62%** |
| Person has episodes of disorganized speech | Daily functioning | Bodily functions= 18%  Daily functioning = 18%  Participation = 5%  **Mental wellbeing= 56%**  No Pillar of Best Fit= 3% |
| Person's decision making abilities are different compared to 90 days ago (e.g., abilities improved or declined) | Mental wellbeing | Bodily functions= 3%  Daily functioning = 44%  **Mental wellbeing= 54%** |
| Person's ability to be understood (e.g., ability to express or communicate requests, needs, opinion and urgent problems and to engage in social conversation) | Participation | Daily functioning = 15%  Participation = 39%  **Mental wellbeing= 44%**  No Pillar of Best Fit= 3% |
| Person's ability to understand others (i.e., ability to understand verbal information) | Mental wellbeing | Bodily functions= 11%  Daily functioning = 11%  Participation = 16%  Meaningfulness= 3%  **Mental wellbeing= 55%**  No Pillar of Best Fit= 5% |
| Person made negative statements (e.g., "Nothing matters", "What's the use", "Would rather be dead than live this way") | Meaningfulness | Quality of Life = 32%  **Meaningfulness= 37%**  Mental wellbeing= 26%  No Pillar of Best Fit= 5% |
| Person demonstrated persistent anger with self or others (e.g., easily annoyed, anger at care received) | Mental wellbeing | Daily functioning = 3%  Quality of Life = 32%  **Mental wellbeing= 53%**  No Pillar of Best Fit= 13% |
| Person demonstrated unrealistic fears (e.g., fear of being abandoned, being left alone or being with others) | Mental wellbeing | Participation = 8%  Quality of Life = 32%  **Mental wellbeing= 55%**  No Pillar of Best Fit= 5% |
| Person made repetitive health complaints (e.g., persistently seeking medical attention, incessant concern with body functions) | Bodily functions | **Bodily functions= 45%**  Daily functioning = 8%  Quality of Life = 16%  Mental wellbeing= 21%  No Pillar of Best Fit= 11% |
| Person made repetitive non-health-related concerns (e.g., seeking attention/ reassurance regarding schedules, meals, laundry and relationships) | Daily functioning | Daily functioning = 18%  Participation = 8%  **Quality of Life = 34%**  Meaningfulness= 5%  Mental wellbeing= 24%  No Pillar of Best Fit= 11% |
| Person demonstrated sad, pained, or worried facial expressions (e.g., furrowed brows, constant frowning) | Mental wellbeing | Bodily functions= 8%  **Quality of Life = 40%**  Meaningfulness= 5%  Mental wellbeing= 37%  No Pillar of Best Fit= 11% |
| Person cried or experienced tearfulness | Mental wellbeing | Bodily functions= 3%  Quality of Life = 34%  Meaningfulness= 3%  **Mental wellbeing= 45%**  No Pillar of Best Fit= 16% |
| Person made recurrent statements that something terrible is about to happen | Mental wellbeing | Participation = 3%  Quality of Life = 24%  **Mental wellbeing= 68%**  No Pillar of Best Fit= 5% |
| Person stopped participating in activities of interest | Daily functioning | Daily functioning = 8%  **Participation = 61%**  Quality of Life = 8%  Meaningfulness= 11%  Mental wellbeing= 8%  No Pillar of Best Fit= 5% |
| Person made nonverbal expressions demonstrating a lack of pleasure in life (e.g., anhedonia) | Quality of life | Participation = 5%  **Quality of Life = 50%**  Meaningfulness= 18%  Mental wellbeing= 21%  No Pillar of Best Fit= 5% |
| Person reports little interest or pleasure in activities normally enjoyed | Participation | Daily functioning = 3%  Participation = 21%  Quality of Life = 21%  **Meaningfulness= 29%**  Mental wellbeing= 21%  No Pillar of Best Fit= 5% |
| Person reports feeling anxious, restless or uneasy | Mental wellbeing | Bodily functions= 5%  Quality of Life = 26%  **Mental wellbeing= 66%**  No Pillar of Best Fit= 3% |
| Person reports feeling sad, depressed, or hopeless | Quality of life | Quality of Life = 26%  Meaningfulness= 13%  **Mental wellbeing= 58%**  No Pillar of Best Fit= 3% |
| Person wanders around frequently (e.g., moved with no rationale) | Bodily functions | Bodily functions= 8%  Daily functioning = 21%  Quality of Life = 5%  Meaningfulness= 5%  **Mental wellbeing= 40%**  No Pillar of Best Fit= 21% |
| Person demonstrated verbally abusive behaviour (e.g., threatened others, screamed at others etc.) | Bodily functions | Bodily functions= 8%  Daily functioning = 3%  Participation = 13%  Quality of Life = 11%  **Mental wellbeing= 53%**  No Pillar of Best Fit= 13% |
| Person demonstrated physically abusive behaviour (e.g., others were hit, shoved) | Bodily functions | Bodily functions= 5%  Daily functioning = 8%  Participation = 13%  Quality of Life = 5%  **Mental wellbeing= 40%**  No Pillar of Best Fit= 29% |
| Person demonstrated disruptive or socially inappropriate behaviour (e.g., made disruptive sounds or noises) | Participation | Bodily functions= 5%  Daily functioning = 5%  Participation = 26%  **Mental wellbeing= 37%**  No Pillar of Best Fit= 26% |
| Person demonstrated sexually inappropriate behaviour or disrobed in public | Bodily functions | Bodily functions= 24%  Daily functioning = 13%  Participation = 3%  Quality of Life = 5%  Meaningfulness= 3%  **Mental wellbeing= 37%**  No Pillar of Best Fit= 16% |
| Person resisted care (e.g., taking medications/ injections, ADL assistance) | Daily functioning | Bodily functions= 5%  **Daily functioning = 48%**  Participation = 3%  Quality of Life = 13%  Meaningfulness= 8%  Mental wellbeing= 8%  No Pillar of Best Fit= 16% |
| Person reports feelings of conflict or anger with family/ friends | Mental wellbeing | Participation = 21%  Quality of Life = 26%  Meaningfulness= 3%  **Mental wellbeing= 34%**  No Pillar of Best Fit= 16% |
| Person was fearful of family member or close acquaintance | Participation | Bodily functions= 3%  Participation = 16%  **Quality of Life = 42%**  Mental wellbeing= 26%  No Pillar of Best Fit= 13% |
| Person experienced neglect, abuse, or mistreatment | Quality of life | Daily functioning = 5%  Participation = 3%  **Quality of Life = 66%**  Mental wellbeing= 5%  No Pillar of Best Fit= 21% |
| Person expresses feelings of loneliness | Quality of life | Daily functioning = 3%  Participation = 32%  **Quality of Life = 34%**  Meaningfulness= 5%  Mental wellbeing= 21%  No Pillar of Best Fit= 5% |
| Person's interest in social, religious, occupational or other preferred activities changed in the last 90 days | Participation | Daily functioning = 3%  **Participation = 66%**  Quality of Life = 8%  Meaningfulness= 11%  Mental wellbeing= 11%  No Pillar of Best Fit= 3% |
| Person experienced a major stressful event in the last 90 days (e.g., episode of severe personal illness, death or illness of close member) | Quality of life | Bodily functions= 5%  Participation = 3%  **Quality of Life = 53%**  Meaningfulness= 3%  Mental wellbeing= 26%  No Pillar of Best Fit= 11% |
| Person's ability to walk up a full flight of stairs (e.g., manage 12-14 stairs) | Daily functioning | **Bodily functions= 63%**  Daily functioning = 37% |
| Person's ability to walk around indoors (i.e., how person walks between locations on same floor) | Bodily functions | **Bodily functions= 58%**  Daily functioning = 42% |
| Person's ability to move around (walking or wheeling) indoors | Daily functioning | **Bodily functions= 55%**  Daily functioning = 45% |
| Person's ability to move on and off toilet or commode | Daily functioning | Bodily functions= 40%  **Daily functioning = 61%** |
| Person's ability to move around in bed (i.e., how person moves to/ from lying position, turns from side to side) | Bodily functions | **Bodily functions= 55%**  Daily functioning = 42%  No Pillar of Best Fit= 3% |
| Person's ability to eat and drink (including other means of intake of nourishment by other means such as tube feeding) | Bodily functions | Bodily functions= 45%  **Daily functioning = 55%** |
| Person's primary means of movement indoors | Daily functioning | **Bodily functions= 40%**  **Daily functioning = 40%**  Participation = 3%  Quality of Life = 3%  No Pillar of Best Fit= 16% |
| If using wheelchair, farthest distance person was able to wheel self | Daily functioning | **Bodily functions= 55%**  Daily functioning = 40%  No Pillar of Best Fit= 5% |
| Total hours of physical activity person participated in | Bodily functions | **Bodily functions= 53%**  Daily functioning = 24%  Participation = 11%  Quality of Life = 5%  No Pillar of Best Fit= 8% |
| Number of days person went out of the house | Participation | Bodily functions= 3%  Daily functioning = 11%  **Participation = 53%**  Quality of Life = 11%  Meaningfulness= 5%  No Pillar of Best Fit= 18% |
| Person believes he/ she is able to improve level of physical function | Meaningfulness | **Bodily functions= 32%**  Daily functioning = 21%  Participation = 3%  Quality of Life = 5%  Meaningfulness= 29%  Mental wellbeing= 8%  No Pillar of Best Fit= 2% |
| Person's health care provider believes person is able to improve levels of physical function | Bodily functions | **Bodily functions= 32%**  Daily functioning = 21%  Participation = 3%  Quality of Life = 13%  Meaningfulness= 8%  Mental wellbeing= 3%  No Pillar of Best Fit= 21% |
| Person drove car (vehicle) in last 90 days | Quality of life | Bodily functions= 3%  **Daily functioning = 52%**  Participation = 21%  Quality of Life = 11%  No Pillar of Best Fit= 13% |
| Person has been told to limit or stop driving | Quality of life | **Daily functioning = 45%**  Participation = 8%  Quality of Life = 24%  Meaningfulness= 3%  Mental wellbeing= 5%  No Pillar of Best Fit= 16% |
| Person uses urinary collection device (i.e., excluding pads/ briefs) | Daily functioning | **Bodily functions= 61%**  Daily functioning = 34%  Quality of Life = 3%  No Pillar of Best Fit= 3% |
| Person uses pads or briefs for incontinence | Daily functioning | **Bodily functions= 66%**  Daily functioning = 29%  Quality of Life = 5% |
| Person was diagnosed with Alzheimer's disease | Mental wellbeing | Bodily functions= 24%  Daily functioning = 8%  Quality of Life = 3%  Meaningfulness= 3%  **Mental wellbeing= 42%**  No Pillar of Best Fit= 21% |
| Person was diagnosed with dementia other than Alzheimer's | Mental wellbeing | Bodily functions= 21%  Daily functioning = 8%  Quality of Life = 5%  Meaningfulness= 3%  **Mental wellbeing= 47%**  No Pillar of Best Fit= 16% |
| Person was diagnosed with multiple sclerosis | Bodily functions | **Bodily functions= 66%**  Daily functioning = 5%  Quality of Life = 5%  Mental wellbeing= 3%  No Pillar of Best Fit= 21% |
| Person was diagnosed with Parkinson's disease | Bodily functions | **Bodily functions= 61%**  Daily functioning = 3%  Quality of Life = 8%  Mental wellbeing= 8%  No Pillar of Best Fit= 21% |
| Person was diagnosed with quadriplegia | Bodily functions | **Bodily functions= 68%**  Daily functioning = 5%  Quality of Life = 5%  No Pillar of Best Fit= 21% |
| Person was diagnosed with stroke/ cerebrovascular accident | Bodily functions | **Bodily functions= 61%**  Daily functioning = 8%  Quality of Life = 8%  Mental wellbeing= 8%  No Pillar of Best Fit= 16% |
| Person was diagnosed with bipolar disorder | Mental wellbeing | Bodily functions= 16%  Quality of Life = 8%  **Mental wellbeing= 63%**  No Pillar of Best Fit= 13% |
| Person was diagnosed with schizophrenia | Mental wellbeing | Bodily functions= 21%  Quality of Life = 3%  **Mental wellbeing= 63%**  No Pillar of Best Fit= 13% |
| Number of falls experienced by the person in the last 90 days | Daily functioning | **Bodily functions= 53%**  Daily functioning = 37%  Quality of Life = 5%  No Pillar of Best Fit= 5% |
| Person has difficulty moving self to standing position unassisted | Daily functioning | **Bodily functions= 53%**  Daily functioning = 47% |
| Person has difficulty turning around when standing | Daily Functioning | **Bodily functions= 61%**  Daily functioning = 37%  No Pillar of Best Fit= 3% |
| Person has difficulty falling/ staying asleep | Daily functioning | **Bodily functions= 63%**  Daily functioning = 18%  Quality of Life = 5%  Mental wellbeing= 8%  No Pillar of Best Fit= 5% |
| Person experiences excessive sleep that interferes with normal functioning | Daily functioning | **Bodily functions= 55%**  Daily functioning = 26%  Participation = 3%  Quality of Life = 11%  Mental wellbeing= 5% |
| Person experiences fatigue (e.g., inability to complete normal daily activities) | Bodily functions | **Bodily functions= 47%**  Daily functioning = 37%  Participation = 3%  Quality of Life = 11%  No Pillar of Best Fit= 3% |
| Frequency with which person complains or shows evidence of pain | Quality of life | **Bodily functions= 58%**  Daily functioning = 11%  Quality of Life = 24%  No Pillar of Best Fit= 8% |
| Person reports that pain control is adequate to the level of pain experienced | Quality of life | **Bodily functions= 50%**  Daily functioning = 16%  Quality of Life = 29%  No Pillar of Best Fit= 5% |
| Person's conditions/ diseases make cognitive issues, activities of daily living, mood, or behaviour patterns unstable | Daily functioning | Bodily functions= 13%  **Daily functioning = 34%**  Quality of Life = 11%  **Mental wellbeing= 34%**  No Pillar of Best Fit= 8% |
| Person is experiencing acute episode or flare-up of a recurrent or chronic problem | Quality of life | **Bodily functions= 66%**  Daily functioning = 5%  Quality of Life = 11%  No Pillar of Best Fit= 18% |
| Person has end-stage diseases, 6 or fewer months to live | Quality of life | **Bodily functions= 40%**  Daily functioning = 3%  Quality of Life = 34%  Meaningfulness= 8%  No Pillar of Best Fit= 16% |
| Person's self-reported health | Participation | Bodily functions= 29%  Daily functioning = 21%  Participation = 3%  **Quality of Life = 40%**  No Pillar of Best Fit= 8% |
| Person's level of tobacco use (e.g., smokes tobacco daily) | Daily functioning | Bodily functions= 18%  **Daily functioning = 47%**  Quality of Life = 3%  Mental wellbeing= 5%  No Pillar of Best Fit= 26% |
| Person's level of alcohol intake (i.e., number of drinks in any 'single sitting' in last 14 days) | Daily functioning | Bodily functions= 11%  **Daily functioning = 50%**  Quality of Life = 3%  Mental wellbeing= 8%  No Pillar of Best Fit= 29% |
| Person shows signs of dehydration | Bodily functions | **Bodily functions= 68%**  Daily functioning = 24%  No Pillar of Best Fit= 8% |
| Person's fluid intake is less than 1,000ml/ day | Daily functioning | **Bodily functions= 58%**  Daily functioning = 31%  No Pillar of Best Fit= 11% |
| Decrease in amount of food/ fluid usually consumed by person | Daily functioning | Bodily functions= 40%  **Daily functioning = 47%**  Mental wellbeing= 3%  No Pillar of Best Fit= 11% |
| Person ate one or fewer meals (i.e., or at least 2 over the last 3 days) | Daily functioning | Bodily functions= 37%  **Daily functioning = 50%**  Mental wellbeing= 3%  No Pillar of Best Fit= 11% |
| How person consumes food (e.g., normal, only pureed solids, use of abdominal feeding tube) | Daily functioning | **Bodily functions= 50%**  Daily functioning = 37%  Quality of Life = 11%  No Pillar of Best Fit= 3% |
| Person wears denture(s) | Daily functioning | **Bodily functions= 50%**  Daily functioning = 24%  Quality of Life = 3%  No Pillar of Best Fit= 24% |
| Person reports experiencing difficulty chewing | Daily functioning | **Bodily functions= 68%**  Daily functioning = 26%  No Pillar of Best Fit= 5% |
| Person has a history of prior pressure ulcer | Quality of life | **Bodily functions= 68%**  Daily functioning = 5%  Quality of Life = 5%  No Pillar of Best Fit= 21% |
| List of all medications taken by person | No Pillar of Best Fit | Bodily functions= 26%  **Daily functioning = 37%**  Quality of Life = 3%  No Pillar of Best Fit= 34% |
| Person has had blood pressure measured in last year | Participation | **Bodily functions= 42%**  Daily functioning = 29%  Participation = 8%  Quality of Life = 3%  No Pillar of Best Fit= 18% |
| Person has had colonoscopy test in last 5 years | Participation | **Bodily functions= 40%**  Daily functioning = 34%  Participation = 8%  Quality of Life = 3%  No Pillar of Best Fit= 16% |
| Person has had a dental exam in last year | Participation | Bodily functions= 32%  **Daily functioning = 40%**  Participation = 8%  Quality of Life = 3%  No Pillar of Best Fit= 18% |
| Person has had an eye exam in last year | Participation | Bodily functions= 32%  **Daily functioning = 42%**  Participation = 8%  Quality of Life = 3%  No Pillar of Best Fit= 16% |
| Person has had a hearing exam in last 2 years | Participation | **Bodily functions= 40%**  Daily functioning = 37%  Participation = 8%  Quality of Life = 3%  No Pillar of Best Fit= 13% |
| Person received influenza vaccine in last year | No Pillar of Best Fit | **Bodily functions= 42%**  Daily functioning = 29%  Participation = 5%  Quality of Life = 5%  No Pillar of Best Fit= 19% |
| Person has had a mammogram or breast exam in last 2 years (for women) | Participation | Bodily functions= 34%  **Daily functioning = 42%**  Participation = 8%  Quality of Life = 3%  No Pillar of Best Fit= 13% |
| Person received a pneumovax vaccine in last 5 years or after age 65 | No Pillar of Best Fit | Bodily functions= 34%  **Daily functioning = 40%**  Participation = 3%  Quality of Life = 3%  No Pillar of Best Fit= 21% |
| Treatments received or scheduled by person (e.g., chemotherapy, IV medication, wound care) | Daily functioning | **Bodily functions= 42%**  Daily functioning = 29%  Participation = 8%  Quality of Life = 3%  No Pillar of Best Fit= 18% |
| Person received care from a care professional in the last 7 days (e.g., home health aide, home nurse, physical/ occupational therapy, speech-language pathology) | Participation | Bodily functions= 13%  **Daily functioning = 32%**  Participation = 21%  Quality of Life = 5%  No Pillar of Best Fit= 29% |
| Person visited hospital, emergency room, or physician visit in the last 90 days | No Pillar of Best Fit | **Bodily functions= 37%**  Daily functioning = 21%  Participation = 11%  Quality of Life = 5%  No Pillar of Best Fit= 26% |
| Person had an inpatient acute hospital visit with overnight stay in the last 90 days | No Pillar of Best Fit | **Bodily functions= 42%**  Daily functioning = 11%  Participation = 3%  Quality of Life = 8%  No Pillar of Best Fit= 37% |
| Person had an emergency room visit excluding overnight stay in the last 90 days | No Pillar of Best Fit | Bodily functions= 37%  Daily functioning = 13%  Participation = 3%  Quality of Life = 8%  **No Pillar of Best Fit= 40%** |
| Person had a physician or authorized assistant/ practitioner visit in the last 90 days | Participation | **Bodily functions= 26%**  Daily functioning = 18%  Participation = 21%  Quality of Life = 8%  **No Pillar of Best Fit= 26%** |
| Person was physically restrained (e.g., limbs restrained to chair) | Daily functioning | Bodily functions= 5%  Daily functioning = 11%  Participation = 5%  **Quality of Life = 42%**  Meaningfulness= 3%  Mental wellbeing= 8%  No Pillar of Best Fit= 26% |
| Person has a decision-maker for personal care and/or property | Participation | Bodily functions= 3%  Daily functioning = 21%  **Participation = 37%**  Quality of Life = 16%  Mental wellbeing= 8%  No Pillar of Best Fit= 16% |
| Person has an informal helper (e.g., informal caregiver) | Participation | Daily functioning = 32%  **Participation = 42%**  Quality of Life = 18%  No Pillar of Best Fit= 8% |
| Informal care giver lives with person | Participation | Daily functioning = 13%  Participation = 29%  **Quality of Life = 34%**  Meaningfulness= 3%  No Pillar of Best Fit= 21% |
| Person's informal helper(s) is unable to continue caring | No Pillar of Best Fit | Daily functioning = 26%  Participation = 13%  **Quality of Life = 37%**  No Pillar of Best Fit= 24% |
| Person's primary informal helper expresses distress, anger, depression | No Pillar of Best Fit | Daily functioning = 5%  Participation = 16%  Quality of Life = 26%  Mental wellbeing= 13%  **No Pillar of Best Fit= 40%** |
| Person's family or close friends report feeling overwhelmed by person's illness | No Pillar of Best Fit | Bodily functions= 3%  Daily functioning = 8%  Participation = 24%  Quality of Life = 26%  Mental wellbeing= 5%  **No Pillar of Best Fit= 34%** |
| Number of hours of informal care and active monitoring received by person in the last 3 days | Quality of life | Bodily functions= 3%  Daily functioning = 26%  Participation = 13%  quality of Life = 21%  **No Pillar of Best Fit= 37%** |
| Person has limited access to home/ rooms (e.g., unable to climb stairs) | Daily functioning | Bodily functions= 11%  Daily functioning = 37%  Participation = 8%  **Quality of Life = 40%**  No Pillar of Best Fit= 5% |
| Person has access to emergency assistance | Quality of life | Bodily functions= 3%  Daily functioning = 26%  Participation = 16%  **Quality of Life = 42%**  No Pillar of Best Fit= 13% |
| Person is able to have grocery delivered to their home | Quality of life | **Daily functioning = 61%**  Participation = 8%  Quality of Life = 29%  No Pillar of Best Fit= 3% |
| Person has had one or more care goals met in the last 90 days | Quality of life | Daily functioning = 26%  Participation = 3%  **Quality of Life = 40%**  Meaningfulness= 18%  No Pillar of Best Fit= 13% |
| Person's overall self-sufficiency has changed significantly in the last 90 days | Daily functioning | Bodily functions= 11%  **Daily functioning = 48%**  Quality of Life = 24%  Meaningfulness= 5%  No Pillar of Best Fit= 13% |
| Time since event or problem that led to person's deterioration | No Pillar of Best Fit | Bodily functions= 18%  Daily functioning = 5%  Participation = 3%  Quality of Life = 24%  Mental wellbeing= 3%  **No Pillar of Best Fit= 47%** |
| Person's residential/ living status after discharge (e.g., private home, assisted living, hospital/ unit, care facility) | No Pillar of Best Fit | Daily functioning = 16%  Participation = 3%  **Quality of Life = 63%**  No Pillar of Best Fit= 18% |
