# Supplementary material for: Mapping a comprehensive assessment tool to a holistic definition of health for person-centred care planning in home care: a modified eDelphi study
Source: BMC Health Serv Res. 2023 Nov 16;23:1268. doi: 10.1186/s12913-023-10203-5 (PMC10655331; doi:10.1186/s12913-023-10203-5)
Supplement: Supplementary file 2 — Supplemental File 2: Summary of Proposed Assessment Elements for Underrepresented My (i.e., Meaningfulness and Participation) Positive Health Pillars *note top bolded row is summary statement of all similar grouped suggestions below [file 12913_2023_10203_MOESM2_ESM.docx]

**Supplemental File 2: Summary of Proposed Assessment Elements for Underrepresented Pillars (i.e., Meaningfulness and Participation) for Positive Health *note top bolded row is summary statement of all similar grouped suggestions below**

| **MEANINGFULNESS** | | | |
| --- | --- | --- | --- |
| **Person has thriving social support networks or feels included as part of a community.****‡** | **Person's ability to volunteer, make positive impact in their communities, or feels part of something bigger** | **Person practices mindfulness, is self-aware, or has a spiritual life** | **Person has goals and purpose (i.e., work, career, vocations, occupations)** |
| Interactions with others | Participation in social projects (donating to nonprofits, participating as volunteer, taking part in efforts to improve health care system) | Persons pursues interests in their spirituality | Person reports having goals |
| Spends time with close relationships | Identifying resources in the community that could offer support to enable a meaningful life | Person practices self-care and values self | Person has hobbies/work that give satisfaction |
| Person reports feeling connected to/belongs to a social group (e.g., has several close friends; looks forward to interacting with people) | Giving to/supporting others | Mood and demeanor | Person regularly sets daily goals or works on areas where they want to make achievements; |
| Having a support system/network that enables the pursuit of ideals | Person reports feeling they are not needed or making a difference / | Engagement in spiritual activities | Having goals for life |
| Person reports feeling well connected and engaged (e.g., low feelings of loneliness) | Need to feel engaged and find passions. | Self-confidence; spirituality and purpose in life | Has sources of entertainment, vocation or employment, |
| Having/making friends, making plans with family, having plans for the future | Active volunteering | Persons works on being mindful and self-aware in daily activities | Person pursues their passions in life (hobbies; sports; creative efforts, etc.) |
| Maintaining friendships, making new friendships and relationships, inclusiveness | Registration in ongoing education program or cultural pursuits including occasional classes or lectures | Self-reflection, spirituality, enjoyment, able to enjoy activities alone or with others | Person felt joy from activities (reading book, listening to music) |
| Observed social interactions | Person reports engagement in community (e.g., volunteering, social groups) | Spiritual life |  |
| Member of social groups (e.g., religious, sports, hobbies) | Engagement in community service | Inherent hope-believing things can be better. | Change in occupations |
| Enjoys Social activities, leisure activities or hobbies | Feelings of being appreciated, of meaning to others. | Ability to know themselves | Economic realities |
| Religious or cultural beliefs/ affiliations, membership in groups | Community resources | Religious or cultural beliefs/ affiliations, membership in groups | Having goals for health care |
| Expresses interest in activities and social interactions | Ability to understand and respond to surroundings |  | Having activities that bring joy |
| Communicates openly with staff and visitors |  |  | Person actively seeking their life's purpose |
| Ability to know and respond to people |  |  | Person has purpose for getting up in morning |
| Ability to understand people |  |  |  |
| Time spent out of room/in activities |  |  |  |
| Having feelings of fulfillment in the care and in social life. |  |  |  |
| **PARTICIPATION** | | | |
| **Person's social and demographic information** | **Person has thriving social support networks or feels included as part of a community. ‡** | **Person's ability to engage in activities of interest** | **Person's emotional health and wellbeing** |
| Access to inclusive activities (including race, gender, orientation etc.),   Having financial ability to access services and activities | Number of social interactions per day/ number of social contacts per week/ membership in club, group or organization, | Participation in exercise program | Having pets |
|  | Person's ability to interact with people around | Person expressed interest in increasing number of activities with others | Self-care |
|  | Person has a network of individuals that they regularly interact with. | Person does not have any hobbies |  |
|  | Person's ability to remember people around | Social activities, meaningful activities or opportunities to participate |  |
|  | Being engaged, enjoying being part of a group, contributing to group | Person is engaged in civic activities |  |
|  | Attendance of event (plays, classes, community activity) | Being with others or finding interests and be engaged in an interest. |  |
|  | Social interactions | Regular activities (daily, weekly, monthly, etc.), regular responsibilities, leisure activities, productivity, engagement, opportunities for engagement, collaboration |  |
|  | Person is involved in meaningful activities and enjoys being around other people. | Person maintains a calendar to track commitments and appointments. |  |
|  | Network analysis of supports in place |  |  |
|  | Access to social activities; extent of meaningful relationships |  |  |
|  | Person involved in organized social clubs, adult day programs etc. |  |  |
|  | Person made plans for get together (virtual or in person) |  |  |
|  | Person has good relationship with family, as well as informal carers and professional care givers. |  |  |
|  | Person communicates or socializes with other people |  |  |
|  | Number of visits received if living in LTC |  |  |
|  | Access to local and surrounding communities, support systems, , levels of communities |  |  |
|  | Person spends lots of time on computer |  |  |
|  | Person's ability to replicate actions of people around |  |  |
|  | Person is an informal helper within their community |  |  |

**‡**This assessment element was suggested under both Meaningfulness and Participation domains
