# Supplementary material for: Mapping a comprehensive assessment tool to a holistic definition of health for person-centred care planning in home care: a modified eDelphi study
Source: BMC Health Serv Res. 2023 Nov 16;23:1268. doi: 10.1186/s12913-023-10203-5 (PMC10655331; doi:10.1186/s12913-023-10203-5)
Supplement: Supplementary file 3 — Supplemental File 3: List of Proposed Assessment Elements and Proposed Additional Descriptors for Underrepresented My Positive Health domains and non-consensus assessment elements, respectively [file 12913_2023_10203_MOESM3_ESM.docx]

**Supplemental File 3: List of Proposed Assessment Elements and Proposed Additional Descriptors for Underrepresented My Positive Health domains and non-consensus assessment elements, respectively**

| **Supplemental File 3a: List of Proposed Assessment Elements for Underrepresented My Positive Health domains** | | | | | | |
| --- | --- | --- | --- | --- | --- | --- |
|  | **Meaningfulness** | | | **Participation** | | |
| 1 | **Person has thriving social support networks or feels included as part of a community.‡** | | | Person's social and demographic information | | |
| 2 | Person's ability to volunteer, make positive impact in their communities, or feels part of something bigger | | | **Person has thriving social support networks or feels included as part of a community.** ‡ | | |
| 3 | Person practices mindfulness, is self-aware, or has a spiritual life | | | **Person's ability to engage in activities of interest** | | |
| 4 | **Person has goals and purpose (i.e., work, career, vocations, occupations)** | | | Person's emotional health and wellbeing | | |
| **Supplemental File 3b: Proposed Additional Descriptors for non-consensus assessment elements** | | | | | | |
|  | **Bodily functions** | **Daily functioning** | **Participation** | **Quality of Life** | **Meaningfulness** | **Mental wellbeing** |
| 1 | **Being able to move about/get around** (1/1) | **Being able to independently complete daily tasks** (1/1) | **Having support networks (e.g., caregiver)** (1/1) | **Having access to care and services** (5/7) | Being self-aware (0/1) | **Having a positive state of mind** (1/1) |
| 2 | Feeling improvement is possible (0/1) | **Being able to complete routine health check-ups** (4/6) |  |  |  |  |
| 3 | **Prognosis of illness/ condition** (1/2) |  |  |  |  |  |
| 4 | **Feeling stable in health status** (1/2) |  |  |  |  |  |
| 5 | **Taking steps to prevent illness/ disease** (1/6) |  |  |  |  |  |

‡This assessment element was suggested under both Meaningfulness and Participation domains

Statements in **bold** reflects the assessment elements and additional descriptors that reached consensus at the end of stage 2

For the additional descriptors, the numbers in parentheses reflect the number of times the additional descriptor reached consensus relative to the number of non-consensus elements from Stage 1 for which that descriptor was proposed [e.g., (1/2) means that the descriptor was suggested for 2 of the 12 non-consensus elements from stage 1, but only reached consensus in one]
